# The Influence of Food Production Experience on Dietary Knowledge, Awareness, Behaviors, and Health among Japanese: A Systematic Review

**DOI:** 10.3390/ijerph17030924

**Published:** 2020-02-02

**Authors:** Daisuke Machida, Osamu Kushida

**Affiliations:** 1 Department of Health and Nutrition, Faculty of Health and Welfare, Takasaki University of Health and Welfare, Takasaki, Gunma 370-0033, Japan; 2 Department of Nutrition and Life Sciences, School of Food and Nutritional Sciences, University of Shizuoka, Yada, Suruga-ku, Shizuoka 422-8526, Japan; kushida@u-shizuoka-ken.ac.jp

**Keywords:** agricultural experience, community garden, school garden, gardening, dietary knowledge, dietary behavior, dietary awareness, health, well-being, systematic review

## Abstract

This study aimed to reveal the influence of food production experience on dietary knowledge, dietary awareness, dietary behaviors, and health among Japanese populations. We conducted a systematic review of articles published between January 2000 and September 2018 (PROSPERO registration number: CRD42019117163) using the following four databases: PubMed, Web of Science, CiNii, and ICHUSHI. The search formulas were created by combining search terms “agricultural experience,” “garden,” “diet,” “food,” “physical activity,” “health,” and “well-being.” The articles were identified by titles, abstracts, and whole texts. We evaluated the content of the articles that met the adoption criteria. We included original articles from peer-reviewed scientific journals, articles written in Japanese or English, observational or interventional studies with statistical analyses, and articles targeting general Japanese people (except for those targeting patients or disabled) to examine the relationship according to the purpose of this review. Nineteen articles met the study criteria, including 10 cross-sectional studies, one retrospective study, seven pre–post studies, and three non-randomized controlled trials. Two studies combined multiple research designs. Thus, food production experiences were suggested to have a positive influence on dietary knowledge, dietary awareness, food preference, dietary behaviors, and mental health among the Japanese. However, the overall quality of the included studies was low. Further verification with randomized controlled trials and prospective cohort studies is required.

## 1. Introduction

The Third Basic Program for *Shokuiku* (food and nutrition education) Promotion formulated by the Japanese Ministry of Agriculture, Forestry, and Fishery sets the basic policies with target values and proposes measures for *Shokuiku* promotion between 2016 and 2020 [1]. This program has a goal of increasing the percentage of people (households) who have experienced work in agriculture, forestry, and fishery [1]. These food production experiences have the purposes of increasing the awareness and understanding of food and diet. However, there is not sufficient scientific evidence of whether this goal is valid. Therefore, it is necessary to scientifically verify what type of effect is expected by the promotion of these food production experiences.

On a global scale, multiple systematic reviews and quick scans have discussed the associations between food production experiences and dietary knowledge, awareness, behavior, and health [2,3,4,5,6,7,8,9,10,11,12,13]. Studies have considered urban gardens [2,3], community gardens [4,5,6], school gardens [7,8,9], garden-based interventions [10,11,12], and community supported agriculture [13] as interventions or exposures. They have included participants in broad life stages such as children, adults, and the elderly. These previous studies noted the following positive effects: dietary knowledge and awareness [2,7,8,9,12], fruit and vegetable preference [7,8,9,11,12], healthy dietary behaviors [2,3,4,5,6,7,8,9,10,11,12,13], physical activity [3,4,7], mental health [3,4,5], and social well-being [3,4,5].

Several systematic reviews on food production experiences have included studies targeting Japanese populations [14,15]. For example, a systematic review of the relationship between vegetable gardening and vegetable intake included two studies of Japanese populations, both of which have confirmed positive associations [14]. In addition, a meta-analysis including two studies of Japanese populations that examined the relationship between gardening and health concluded that gardening had positive effects on health [15]. However, we cannot find a systematic review that clarifies the influence of food production experiences on dietary knowledge, dietary awareness, dietary behavior, and health focusing on the Japanese population. The articles adopted in recent systematic reviews were mostly conducted in the United States and Europe. There are some differences in culture and social environment between Japan and these countries. In other words, sufficient evidence to support the goal set by the Ministry of Agriculture, Forestry, and Fisheries has not been collected [1].

Here, we conducted a systematic review of recent studies among Japanese populations about the relationships between food production experiences and dietary knowledge, dietary awareness, dietary behaviors, and health to collect Japanese evidence on this subject. The food production experiences defined in this study include daily food production (such as home gardening and school gardening) and food production experiences on farms. In addition, we included not only agriculture, but also fishery and forestry (the production of mushrooms and wild plant shoots was classified as forestry in Japan [16]). However, we excluded studies about professional farmers and non-food production agricultural experiences (such as growing flowers or trees). Additionally, dietary awareness includes food interests, food preference, and the feeling of having no leftovers, which are predisposing factors for dietary behaviors, similar to dietary knowledge [17]. Dietary behaviors include actual behavior, such as dietary intake, meal preparations, and changing of taste. The World Health Organization [18] defines health as follows: “a state of complete physical, mental and social well-being and not merely the absence of disease or infirmity.” In the study referred to above [18], physical health (e.g., body mass index (BMI) and blood pressure), mental health (including not only bad mental symptoms but also positive or negative emotions, such as mood), and social well-being (e.g., social cohesion) was defined as health. Subjective health, a measure of overall health and known to be related to mortality [19], was also adopted as a component of health. Because quality of life and well-being interact with health, or are often used synonymously with health, they were included in this review as indicators of overall health and subjective health. In addition, physical activity, a factor affecting health as well as diet [17], was included in this review.

## 2. Materials and Methods

### 2.1. Search Strategy

This research was reported according to the Preferred Reporting Items for Systematic Reviews and Meta-analyses Statement (PRISMA) [20]. We conducted literature searches from October 29 to December 19, 2018, using the following four databases: PubMed, Web of Science, Citation Information by National institute of informatics (CiNii), and Igaku Chuo Zasshi (ICHUSHI). We tried to collect comprehensive Japanese evidence by searching for articles written in Japanese using two domestic databases (Cinii and ICHUSHI). We set PI(E)CO as follows: P (patient): Japanese; I(E) (intervention (exposure)): having food production experience; C (comparison): not having food production experience; O (outcome): dietary knowledge, dietary awareness, food preference, dietary behavior, physical activity, and health conditions. Based on the PI(E)CO method, search terms were selected, and search formulas used in each database were created (Supplementary Table S1). The review plan was registered under the International Prospective Register of Systematic Reviews (PROSPERO; registration number: CRD42019117163) [21].

### 2.2. Screening

The inclusion criteria for the articles were as follows: articles with original data in peer-reviewed scientific journals issued by an academic association; written in Japanese or English; observational or interventional studies in which statistical analyses were performed; targeted general Japanese people aged one year old or older and examined relationships between food production experiences and dietary knowledge, dietary awareness, food preference, dietary behaviors, physical activity, or health conditions. The publication date range was set from January 1, 2000, to September 30, 2018. We excluded studies that targeted patients or disabled people and focused on professional farmers and gardeners. Based on these criteria, the titles and abstracts were scrutinized (primary screening). In addition, more articles that met the criteria were added by a manual search of the reference lists of the identified articles and a related book [22]. Then, the text of the selected documents was read carefully (secondary screening). The above process was conducted independently by the first author and the second author. If there were any differences in the screening result, we resolved it via discussion. Then, we organized the evidence table about the subjects, research methods, and results, and adjusted the variables according to the articles.

### 2.3. Assessment of Research Quality

We conducted an assessment of the risk of bias using the Study Quality Assessment Tools from the National Institute of Health National Heart, Lung, and Blood Institute [23]. Specifically, the Quality Assessment Tool of Controlled Intervention Studies (14 items) for intervention studies with controlled groups, the Quality Assessment Tool for Before–After (Pre–Post) Studies with No Control Group (12 items) for intervention studies with no control groups, and the Quality Assessment Tool for Observational Cohort and Cross-Sectional Studies (14 items) for observational studies were used. First, each item was classified as YES, NO, or OTHER (cannot determine: CD, not applicable: NA, not reported: NR). Then, we calculated the percentage of YES responses for the total number of items excluding NA (the higher the ratio, the lower the risk of bias). The studies with a YES percentage of 32% or less were ranked as “poor,” those with 33% to 65% as “fair,” and those with 66% or more as “good,” according to a previous systematic review [2]. This assessment was conducted by the first author, and the second author checked the results of the assessment. If there were any differences in the assessment results, we resolved them via discussion.

## 3. Results

### 3.1. Article Selection

**Figure 1:**
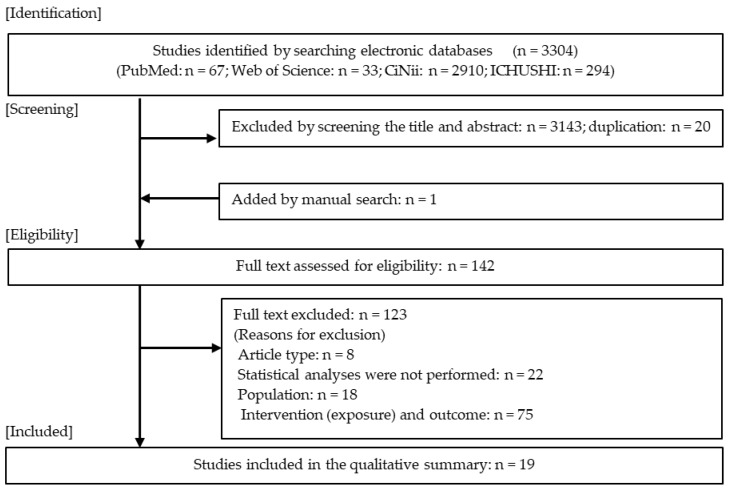
Flowchart of the study selection process.

The flowchart of study selection is shown in Figure 1. From the database search, a total of 3304 articles (PubMed: 67, Web of Science: 33, CiNii: 2910, ICHUSHI: 294) were identified. We excluded 3163 cases in the primary screening. In addition, one article was added by manual searching. Secondary screening was performed for the remaining 142 articles, and 123 were excluded. Finally, 19 articles were included [24,25,26,27,28,29,30,31,32,33,34,35,36,37,38,39,40,41,42].

### 3.2. Outline of Articles

We present the evidence table of the included articles in Table 1. All of the 19 articles (10 cross-sectional studies, one retrospective study, seven pre–post studies, and three non-randomized controlled trials) were conducted in Japan. We grouped the articles according to age of the participants, as described below.

Three of the 19 articles focused on preschoolers [24,25,26], including one cross-sectional study [24], one pre–post study [26], and one combined non-randomized controlled and pre–post study [25]. The interventions or exposures in these studies included one study assessing tomato gardening experience at home [24] and two studies assessing growing and harvesting vegetables (tomatoes or eggplant) in kindergarten [25,26]. The results showed positive relationships between the exposures and a preference for grown vegetables [24,25], dietary awareness [24,25], and dietary behaviors [24,25,26]. However, the evaluation methods were essentially self-administered questionnaires without demonstrated validity or reliability of the outcome indicators.

Among the 19 articles, nine targeted elementally and junior high school students [27,28,29,30,31,32,33,34,35]. Four were cross-sectional studies [28,29,30,31], two were pre–post studies [32,34], two were non-randomized controlled studies [33,35], and one was a combined non-randomized controlled and cross-sectional study [27]. The interventions or exposures in these studies included eight studies assessing agriculture or gardening experiences via school programs [27,28,29,30,31,32,33,35]. These included both in-school and out-of-school programs. One study considered not only school programs but also vegetable growing at home and agricultural experiences on trips as the exposures [31]. One study treated an agricultural experience as an intervention; however, there was no clear statement as to whether it was a school program [34]. The results of five studies showed a significant association with good dietary awareness among students with food production experience [27,28,29,30,35]. One study showed significantly more dietary knowledge and better behavior related to diet [31]. Two studies confirmed good changes in mood [32,34], and one study showed significant improvement in the sense of taste [33]. Six of the nine studies described the validity and/or reliability of the outcome measures [28,29,30,32,33,34].

Three of the 19 articles targeted university and junior college students [36,37,38]. One was a retrospective study [36], and two were pre–post studies [37,38]. The interventions or exposures in these studies included one study that assessed agricultural experience from elementary school to high school [36] and two studies that assessed horticulture works such as sowing seeds, weeding, and plowing [37,38]. The results of one study showed a positive relationship between agricultural experience and dietary awareness and dietary knowledge [36]. However, there was no description about the validity or reliability of the outcome indicators [36]. Another study showed that diastolic blood pressure was significantly increased, and pulse rate was significantly decreased soon after sowing vegetable seeds [37]. There was no significant change in systolic blood pressure [37]. Another study confirmed good changes in mood as a whole soon after sowing [38]. Additionally, there was a significant increase in feeling pleasure soon after weeding and sowing [38]. However, a significant decrease in relaxation soon after weeding and plowing was also shown [38]. This study described the validity of the outcome measures [38].

Four of the 19 articles targeted adults and the elderly [39,40,41,42]. All were cross-sectional studies [39,40,41,42]. The exposures in three of these studies were community gardens [39,40,42], and one of these studies additionally assessed agricultural experience on a farm [42]. Another study assessed the number of types of cultivation activities being performed [41]. The results of one study showed a significant positive relationship between participation in community gardens or agricultural experience on farms and almost all survey items of dietary awareness (20/21 items) [42]. Another study showed significant positive relationships between the number of types of cultivation activities and opportunities to obtain vegetables and dietary diversity [41]. Yet another study revealed significant positive relationships between urban community gardening and vegetable intake, perceived general health, subjective health complaints, general mental health, and social cohesion [39]. However, community gardeners drink more frequently [39]. BMI and physical activity were not associated with community gardening [39]. Conversely, community gardening in a suburban area was significantly associated with a greater amount of physical activity [40]. Additionally, vegetable intake, perceived general health, and social cohesion were not related with community gardening in a suburban area [40]. There was no significant relationship between community gardening and BMI in a suburban area, similar to urban community gardening [40]. In three of the four studies, outcome measures that were evaluated for reliability and/or validity were used [39,40,41].

### 3.3. Study Quality

Of the 10 cross-sectional studies and one retrospective study, 10 [24,28,29,30,31,36,39,40,41,42] had a “Fair” study quality and one [27] was “Poor.” Of the seven pre–post studies, one [32] had a “Good” study quality and six [25,26,27,34,37,38] were “Fair.” Of the three non-randomized controlled trials, one [25] had a “Fair” study quality and two [33,35] were “Poor.” The details of the assessment are shown in Supplementary Table S2.

## 4. Discussion

In this systematic review, we summarized the relationships between food production experiences and dietary knowledge, dietary awareness, dietary behaviors, and health among Japanese populations. Nineteen articles were assessed in this review [24,25,26,27,28,29,30,31,32,33,34,35,36,37,38,39,40,41,42] that evaluated agricultural experiences, such as gardening, as interventions and exposures. No studies assessed food production experiences that could be classified as fishery or forestry. Therefore, in the future, the relationship between fishery or forestry experiences and dietary knowledge, dietary awareness, dietary behaviors, and health must be examined. This study has provided much clarification about the relationship between agricultural experiences and dietary knowledge, dietary awareness, dietary behaviors, and health. We discuss this subject below.

### 4.1. Dietary Knowledge and Awareness

For preschoolers, positive relationships were shown between agricultural experiences and dietary awareness [24,25]. Similarly, positive effects on food preference were suggested [24,25]. The results were consistent in the two studies assessing this age group. Western studies have also reported improved dietary awareness among preschoolers who participate in gardens [9,10,11]. There may have been an increase in interest and preference due to increased exposure [43]. Although two such studies were assessed in this review, one [24] was the baseline analysis of the other study [25]. Therefore, the participants were overlapping. Additionally, research using assessment tools that have been verified for validity and reliability is desirable. In the future, it will be necessary to reexamine whether there is a similar trend in other regions in Japan. Moreover, no studies examined the relationship between agricultural experiences and dietary knowledge.

School children also displayed positive relationships between agricultural experiences and dietary awareness [27,28,30,35]. In particular, the index of gratitude for food used in two studies has been verified for reliability and validity and is credible [28,30]. Additionally, positive relationships between agricultural experiences and dietary knowledge were confirmed [31]. However, only one study with this finding has been reported, and the validity of the indicators has not been shown, so further verification is necessary in the future among Japanese populations.

No studies examined the relationship between agricultural experiences and dietary awareness in university and junior college students. One study suggested a positive association with dietary knowledge [36]. Additionally, in adults and the elderly, one study showed a positive association between agricultural experiences and dietary awareness [42], and no studies examined the association between agricultural experiences and dietary knowledge. There was no description about the validity of the outcome indicators [36,42]. It has become clear that there are few studies in Japan that use dietary knowledge and awareness as outcomes among these life stages. Western studies have identified a relationship between urban gardening and increased dietary awareness among adults [2]. Overall, further research is desired in the future among these life stages in Japan.

### 4.2. Dietary Behaviors

For subjects ranging in age from preschoolers to college students, we confirmed a positive relationship between agricultural experiences and dietary behavior in multiple studies [24,25,26,29,31,33,36]. Both an increased intake of harvested crops [24,26] and relationships with the following various dietary behaviors were reported: helping with shopping and meal preparation [25,31], eating balanced meals [25,36], not leaving food leftover [29], and improvement of sense of taste [33]. We assume that the changes in dietary behaviors occurred due to changes in the determinants of dietary behavior such as dietary knowledge and awareness [44]. Agricultural experience not only increases interest and preference for the cultivated crop and increases its intake, but increased dietary interest may also lead to a positive change in overall dietary behavior. However, only two studies using an outcome indicator demonstrated validity or reliability [29,33]. Additionally, studies conducted in the United States have reported that the combination of gardening experience in early childhood and gardening during college, and continued gardening for two years in college students, have a further positive influence on fruit and vegetable intake [45,46]. In the future, such long-term research must be conducted in Japan.

For adults and the elderly, a positive relationship between cultivation and vegetable availability and dietary diversity was found in an urban area [41]. Higher vegetable availability increases vegetable intake frequency, which is believed to have resulted in improved dietary diversity. In addition, the results of the relationship between community gardening and fruit and vegetable intake were not consistent in the two studies assessing this topic [39,40]. One study showed a positive relationship [39], whereas the other did not [40]. This may be due to differences in the survey areas. In urban areas, community gardening is related to fruit and vegetable intake because it becomes an effective route to obtain fruits and vegetables [39]. However, in suburban areas, home gardening is also an important route to obtain fruits and vegetables in addition to community gardening [40]. Additionally, suburban residents often obtain fruit and vegetables from neighbors [47]. Thus, there are many factors that affect fruit and vegetable intake, and it seems there was no positive relationship between community gardening and fruit and vegetable intake in the suburban area. However, in the United States, a positive relationship between community gardening and fruit and vegetable intake has been reported in both rural and urban areas [48,49,50,51]. In addition, after conducting this systematic review, new Japanese articles were published that reported a positive relationship between vegetable cultivation or home/community gardening and vegetable intake [47,52]. The study about vegetable cultivation evaluated residents living in rural, suburban, and urban areas and adjusted for the random effects of the areas in the analysis [47]. The other study was a web survey on community gardening; thus, we cannot grasp the geographical conditions in which the respondents live [52]. Generally, a positive relationship between agricultural experiences and fruit and vegetable intake among adults and the elderly was shown. However, further research will be necessary for the consideration of regional differences such as the accessibility of fruit and vegetables. Moreover, we must note that the evaluation method of fruit and vegetable intake in the studies did not demonstrate sufficient validity or reliability [39,40,47,52].

### 4.3. Physical Activity

Physical activity was assessed in two studies in adults and the elderly [39,40]. However, the results were not consistent. Furthermore, in the latest report, a positive relationship between community gardening and physical activity was reported [52]. We think this may be due to the differences between urban and suburban areas. Individuals tend to travel by car in suburban areas, and physical activity levels tend to be low overall. Therefore, community gardening is a good opportunity for physical activity. Conversely, in urban areas, many individuals travel by walking, and the overall physical activity level is high. Therefore, community gardening does not appear to contribute to physical activity in urban areas. Of course, we must consider that only one study used an indicator with demonstrated validity and reliability [40]. Additionally, we could not identify any reports that examined the influence on physical activity in studies other than among adults and the elderly. In the United States, a study on elementary school children reported a positive association between physical activity and school garden participation [53]. In the future, the relationship between gardening and physical activity should be studied among Japanese children.

### 4.4. Overall Health

Subjective health was also reported only in adults and the elderly, and these results were inconsistent [39,40]. The evaluation methods were the same in two studies, which used a single question scored on a five-point scale [39,40]. Gender differences may be responsible for the discrepancy in results. One study included only men, and there was no significant relationship between subjective health and community gardening [40]. A German study showed that gardening moderated the relationship between self-rated health and social exclusion only among women, not men [54]. Women are likely more involved in social activities during gardening than men, or conversely, women replace or supplement social contact with plants, and as a result, subjective health may be maintained due to a reduction in the perception of social exclusion [54]. Referring to this finding, there is a gender difference in the impact of gardening on subjective health; that is, the effect may be significant in women. In addition, a study in the United States reported a positive relationship between self-rated health and community gardening via collective efficacy using a path analysis [55]. However, such a path analysis has not been conducted in Japanese studies. Thus, further studies about the relationship between agricultural experiences and subjective health, and the underlying mechanism of the relationship are necessary in Japanese populations.

### 4.5. Physical Health

BMI was also reported only in adults and the elderly; these results were consistent in that community gardening was not significantly associated with BMI [39,40]. A study in the United States confirmed an inverse relationship between BMI and community gardening among adults [56]. The average BMI of participants in the U.S. study [56] was clearly higher than that of a study in Japan [40]. Another study in Japan did not report a representative value for BMI [39], but it is likely similar to that of the first Japanese study [40]. Therefore, there seems to be an inverse relationship between gardening and BMI if a group with a high BMI is assessed. In addition, a study on children in New Zealand reported an inverse relationship between gardening and BMI [57]. The relationship between gardening and BMI among children must be examined in Japan.

In junior college students, the relationships between sowing seeds and blood pressure and pulse were reported [37]. The mechanism of this relationship seems to be due to exercise and stress load, but this is not clarified in the study [37]. The National Heart, Lung, and Blood Institute recommends 30–45 min of physical activity (gardening) to reduce blood pressure [58]. This study examined changes in blood pressure and pulse before and soon after sowing seeds [37]. However, the medium to long-term reduction of blood pressure due to habitual gardening must be examined.

### 4.6. Mental Health

Two studies of elementary and junior high school students showed reductions in anxiety and anger due to agricultural experiences [32,34]. Furthermore, in junior college students, improvements in fatigue and overall mood after sowing and improvements in pleasure feelings after weeding and sowing were observed [38]. However, the sense of relaxation decreased after weeding and plowing [38]. In a study of adults and the elderly, community gardeners had better mental health than non-community gardeners [39]. In these studies, well-developed indicators were used to evaluate mental health [32,34,38,39]. Previously, a meta-analysis with gardening as an intervention (exposure) reported a positive association with reduced anxiety and depression [19]. Overall, moderate agricultural activity that is not too burdensome may have positive impacts on mental health.

### 4.7. Social Well-Being

There were two reports on community gardening and social cohesion for adults and the elderly; however, the results were not consistent [39,40]. The scales used in these studies were not the same, but both of the scales had been developed in previous studies [39,40]. In addition, an article published after this systematic review showed a positive association between gardening and social ties [52]. This discrepancy may be due to gender differences. The studies involving both men and women showed a positive relationship [39,52]. However, the study involving only men did not show a positive association [40]. As noted above, gardening was shown to moderate the relationship between self-rated health and social exclusion only among women, not men [54]. Therefore, with a focus on the gender difference, further research about the relationship between agricultural experiences and social well-being is necessary in Japan.

### 4.8. Limitations

This study has some limitations. First, the overall study quality was not high. There were no randomized controlled trials or prospective cohort studies. There were some non-randomized controlled studies and one retrospective study, but the study quality was not good. The only article that was assessed as having good study quality was a pre–post study. Therefore, more high-quality studies are necessary in Japan. In addition, the possibility of publication bias cannot be denied. Here we only targeted articles with original data in peer-reviewed scientific journals issued by academic associations. Thus, we may need to explore gray literature to avoid publication bias and to increase completeness. However, we might have avoided publication bias by using documents written in Japanese identified via domestic databases. Moreover, although only Japanese people are targeted in this study, it is possible that new knowledge may be obtained by evaluating research in East Asian countries that have similar social contexts as Japan.

## 5. Conclusions

The results suggest that there is a positive influence of agricultural experiences on dietary knowledge, dietary awareness, dietary behaviors, and mental health among Japanese populations. Accordingly, it was suggested that promoting agricultural experiences could contribute to improving the diet and mental health of Japanese people. However, there were no randomized controlled trials or prospective cohort studies, and the overall quality of the studies was not high. In the future, considering the differences in intervention types and cultivation methods, further verification with randomized controlled trials and prospective cohort studies is required among Japanese populations. In addition, there have been no reports showing the influence of fishery and forestry experience on dietary knowledge, dietary awareness, dietary behaviors, and health, so we must examine these influences.

## Figures and Tables

**Table 1:** Evidence table of food production experiences and dietary knowledge, dietary awareness, dietary behaviors and health.

Author (Year) [Reference Number]	Settings	Participants	Design/Intervention Term	Intervention (Exposure)	Outcome Investigated/Measurement Method	Results about the Benefits of Food Production Experience	Adjusted Variables	Quality Rating *
**Kida H. et al. (2018) [24]**	Sapporo city, Hokkaido, JPN	335 children aged 3 to 4 years old in 6 kindergartens	Cross-sectional study	Tomato gardening at home	Preference of tomatoes, tomato eating habits, interest in foods (5 items)/self-administered questionnaire	Children who experienced tomato gardening at home tend to do the following more than children who did not experience tomato gardening: like tomatoes (*p* = 0.001), eat tomatoes without leaving (*p* < 0.001), help prepare food (*p* = 0.043), request tomatoes for food and snacks (*p* = 0.004)	None	Fair
**Kida H. et al. (2016) [25]**	Hokkaido, JPN	221 children aged 3 to 4 years old in 5 kindergartens in a city (intervention group: 144 children in 3 kindergartens; control group: 77 children in 2 kindergartens)	Non-randomized controlled trial and pre–post study/May to September 2012, and follow-up survey in March 2013	Gardening, harvesting, and tasting tomatoes	Unbalanced diet (whether they eat when served something they dislike), preference of tomatoes, interest in foods (4 items)/self-administered questionnaire	At follow-up, the percentage of those who improved their unbalanced diet was significantly higher in the intervention group than in the control group (*p* < 0.036). In the intervention group, unbalanced diet, preference of tomatoes, and interest in foods (2/4 items) improved after the intervention and at follow-up compared with before the intervention (*p* < 0.05). There was no significant change in these outcomes in the control group.	None	Non-randomized controlled trial: Fair Pre–post study: Fair
**Kanno Y. et al. (2011) [26]**	Niigata city, Niigata, JPN	38 children aged 4 years old in a kindergarten	Pre–post study/May to July 2010	Vegetable gardening (gardening, harvesting, cooking, and tasting eggplant)	Vegetable intake at lunch in kindergarten/survey on amounts of leftover eggplant intake at home/self-administered questionnaire (parents)	Stir-fried eggplant with miso (*p* = 0.001), stir-fried eggplant with curry (*p* < 0.001), and another side dish (*p* < 0.001) intake at lunch in kindergarten were increased after the intervention. Eggplant intake at home was not significantly different before and after the intervention (*p* = 0.525).	None	Fair
**Ying G. et al. (2014) [27]**	Fukuoka, JPN	116 elementally school students in a rural town (intervention group: 64; control group: 52)	Pre–post study and cross-sectional study/September 2012 to March 2013	Cultivation and processing of vegetables in class	Dietary awareness (5 items: allotment so that the score of the positive answers is high)/self-administered questionnaire	After the intervention compared with before the intervention, the proportions of students who answered “I have no food I dislike (*p* < 0.05)” and “I usually want to eat a lot of vegetables (*p* < 0.01)” increased. In the comparison between the intervention group and control group after the intervention, the proportions of students who answered “I have no food I dislike (p < 0.05)” and “I usually want to eat a lot of vegetables (p < 0.01)” were greater in the intervention group than in the control group.	None	Pre–post study: Fair Cross-sectional study: Poor
**Taniguchi T. et al. (2010) [28]**	Fukushima, JPN Saitama, JPN	368 fifth-grade elementally school students	Cross-sectional study	Subjective scale of school gardening experience (1 = never to 4 = often)	Feelings of gratitude for food and attitudes toward local products/self-administered questionnaire	In the entire analysis, there were positive correlations between school gardening experiences and feelings of gratitude for food (*p* < 0.05) and attitude toward local products (*p* < 0.01). According to the analyses by prefecture, there were no significant positive correlations with feelings of gratitude for food, but there was a significant positive correlation with attitude toward local products (*p* < 0.01 in both prefectures).	None	Fair
**Taniguchi T. et al. (2010) [29]**	Tokyo, JPN	1994 fifth- and sixth-grade elementally school students in a ward	Cross-sectional study	Subjective scale of school gardening experience (1 = never to 4 = often)	Leftover food behavior score/self-administered questionnaire	There was a significant positive association between school gardening experiences and leftover food behavior score (total: *p* = 0.005; boys: *p* = 0.035, girls: *p* = 0.005).	Preference of vegetables, feeling of waste, outcome expectancies, discipline at home	Fair
**Akamatsu R. et al. (2009) [30]**	Tokyo, JPN	1994 fifth- and sixth-grade elementally school students in a ward	Cross-sectional study	Subjective scale of school gardening experience (1 = never to 4 = often)	Gratitude for food scale score/self-administered questionnaire	Those who answered “often” to the school gardening experience had a significantly higher score of gratitude for food than the others (*p* < 0.001).	None	Fair
**Oura Y. et al. (2009) [31]**	Gunma, JPN	524 fifth- and sixth-grade students from four elementary schools	Cross-sectional study	Schools that were specified as model schools for food and agriculture education, vegetable growing experience at home, agricultural experience on trips	Dietary knowledge (3 items), Dietary behavior (1 item)/self-administered questionnaire	More students in schools that were specified as model schools for food and agriculture education used local vegetables in school lunch (*p* < 0.01) and knew the sweet potato harvest season (*p* = 0.02) than students in other schools. More students with a vegetable growing experience at home used local vegetables in school lunch (*p* < 0.01) and knew local traditional foods (*p* < 0.01) than students who do not have a vegetable growing experience at home. More students who had agricultural experience on trips knew local traditional foods (*p* = 0.02) and more frequently went shopping with family members (*p* < 0.01) than those who did not have agricultural experience on trips.	None	Fair
**Yamada I. (2008) [32]**	Iiyama city, Nagano, JPN	41 fifth-grade students from one elementary school	Pre–post study/8 days in September 2006	Experience of agriculture and rural life as part of class	Profile of mood states/self-administered questionnaire	“A week ago” and “after” the experience, the students’ “nervousness-anxiety” was significantly relieved (*p* < 0.05). “Anger-hostility,” “vitality,” and “fatigue” were not significantly different.	None	Good
**Yoshida T. et al. (2007) [33]**	Hamamatsu city, Shizuoka, JPN	130 first-grade students from 2 elementary schools (intervention school 67; control school: 63)	Non-randomized controlled trial/April 2000 to April 2002	Eating education program (select four dishes, cook by yourself, experience a farm, taste discussion, and observe stools)	Taste discernment ability/the whole-mouth method using Taste Desk (Sanwa Kagaku Kenkyusho CO LTD, Nagoya, Japan)	Compared with the control school, the sensitivity results for sour (*p* < 0.05), bitter (*p* < 0.05), and total score of four tastes (*p* < 0.05) were significantly increased in the intervention school students.	None	Poor
**Yamamoto T. (2008) [34]**	Hokkaido, JPN Niigata, JPN	447 students ranging in age from third grade of elementary school to first grade of junior high school	Pre–post study/unconfirmed	Experience of agriculture and rural life	Psychological evaluation method of experience training for children/self-administered questionnaire	Overall, anger (*p* < 0.01) and anxiety (*p* < 0.01) decreased after the intervention.	None	Fair
**Shimamura M. et al. (2013) [35]**	Nagoya city, Aichi, JPN	599 students in the second grade of junior high school (intervention group: 449; control group: 150)	Non-randomized controlled trial/2008 to 2010	“One pot to one person” cultivation of *Gymnema sylvestre*	Interest in taste and foods/self-administered questionnaire	After the intervention, interest in taste and foods was significantly higher in the intervention group than in the control group (*p* < 0.05).	None	Poor
**Sato K. (2015) [36]**	JPN	590 first- to fourth-grade students at a nursing university (exposed group: 484; control group: 106)	Retrospective study	Agricultural experience from elementary school to high school	Dietary behavior: 7 items, dietary knowledge: 3 items (allotment so that the score of negative answers is high)/self-administered questionnaire	The following items had significantly lower scores in the exposed group: For dietary behavior, “Eat a complete meal of a staple food, main dish, and side dish for dinner (*p* = 0.048),” “I would like to participate in harvest work if there are fields in the university (*p* = 0.009).” For dietary knowledge, “I know about seasonal vegetables (*p* = 0.007),” “I know the word *chisanchisho* (local production for local consumption) (*p* = 0.013),” and “I know the four-group food score table (*p* = 0.015).”	None	Fair
**Tsuchihashi Y. (2010) [37]**	Nishinomiya city, Hyogo, JPN	24 s-grade students in junior college (all women)	Pre–post study/April to July 2005	Sowing vegetable seeds	Blood pressure, pulse rate/automatic digital blood pressure monitor	The diastolic blood pressure was significantly increased (*p* < 0.05) and the pulse rate was significantly decreased (*p* < 0.01). There was no significant change in systolic blood pressure.	None	Fair
**Otake M. et al. (2010) [38]**	Hokkaido, JPN	9 university students	Pre–post study/July to September 2007	Horticulture work (weeding, plowing, and sowing)	Profile of mood states/self-administered questionnaire. Pleasure feeling and relaxation (MCL-S.1)/self-administered questionnaire	The fatigue score (*p* = 0.026) and the overall score (*p* = 0.026) decreased significantly after sowing. There was a significant increase in pleasure feeling after weeding (*p* < 0.05) and sowing (*p* < 0.05) and a significant decrease in relaxation after weeding (*p* < 0.05) and plowing (*p* < 0.05).	None	Fair
**Soga M. et al. (2017) [39]**	Nerima, Tokyo, JPN	332 residents in Nerima-ku (exposed group: 165, control group: 167)	Cross-sectional study	Participation in community gardening	Drinking, vegetable intake, physical activity perceived general health, subjective health complaints, BMI, general mental health (GHQ12), social cohesion/self-administered questionnaire	There was a relationship between good health and participation in community gardens for the following indicators: vegetable intake (*p* < 0.001), perceived general health (*p* < 0.001), subjective health complaints (*p* < 0.05), general mental health (*p* < 0.05), and social cohesion (*p* < 0.001).	None Sex, age, family income, employment status, smoking, drinking, vegetable intake, physical activity	Fair
**Machida D. et al. (2017) [40]**	Gunma, JPN	251 men aged 50–74 years old living in a city (community gardener: 30, vegetable cultivator without community gardener: 91, nongardener: 130)	Cross-sectional study	Participation in community gardening	BMI, self-rated health Fruit and vegetable intake, physical activity, sitting time Social cohesion/self-administered questionnaire	The odds ratio of those performing a large amount of physical activity was 3.00 (1.18–7.64) among community gardeners compared with nongardeners. There was no significant association between the other outcomes and community gardening.	None Age Educational background	Fair
**Amemiya M. (2012) [41]**	Kashiwa city, Chiba, JPN	1612 residents in Kashiwa city aged 20–79 years	Cross-sectional study	Number of types of cultivation activities performed	Opportunity to obtain vegetables, Dietary diversity/self-administered questionnaire	If there were many types of crop cultivation activities, there were more opportunities to obtain vegetables (*p* = 0.01). If there were many types of crop cultivation activities, there was more dietary diversity (*p* < 0.01).	None	Fair
**Noda T. (2007) [42]**	Nerima-ku, Tokyo, JPN	645 residents in Nerima (agricultural experience farm participants: 295, community garden participants: 182, control (non-participants): 168)	Cross-sectional study	Participation in agricultural experience farm, Participation in community gardening	Dietary awareness (21 items)/self-administered questionnaire or interview	Agricultural experience farm participants and community garden participants had significantly higher dietary awareness than the control group (20/21 items: *p* < 0.05).	None	Fair

* The Study Quality Assessment Tools from the National Institute of Health National Heart, Lung, and Blood Institute: 32% or less = “poor,” 33 to 65% = “fair,” 66% or more = “good”.

## Supplementary Materials

The following are available online at https://www.mdpi.com/1660-4601/17/3/924/s1, Table S1: The search formula used for each database, Table S2: Results of bias risk assessment, and PRISMA checklist [59] of this systematic review.
