# Supplementary material for: The Influence of Food Production Experience on Dietary Knowledge, Awareness, Behaviors, and Health among Japanese: A Systematic Review
Source: Int J Environ Res Public Health. 2020 Feb 2;17(3):924. doi: 10.3390/ijerph17030924 (PMC7037670; doi:10.3390/ijerph17030924)
Supplement: Supplementary file 1 [file ijerph-17-00924-s001.zip › Sup_ijerph-703582-final.pdf]

The Influence of Food Production Experience on Dietary Knowledge, Awareness, Behaviors, and Health among Japanese: A Systematic Review

Daisuke Machida and Osamu Kushida

Table S1. Search formulas used in each database.

Database	Search formulas
PubMed	(Japan*) AND (“agricultural experience”[tiab] OR “farming experience”[tiab] OR “farming workshop”[tiab] OR “forestry experience”[tiab] OR “fishery experience”[tiab] OR “rural experience”[tiab] OR “farm stay”[tiab] OR “agritourism”[tiab] OR “food production practice”[tiab] OR “community supported agriculture”[tiab] OR garden*[ti] OR horticultur*[ti] OR harvest*[ti]) AND (diet*[tiab] OR food*[tiab] OR nutri*[tiab] OR vegetable*[tiab] OR fruit*[tiab] OR health*[tiab] OR “exercise”[tiab] OR “physical activity”[tiab] OR “body weight”[tiab] OR “obesity”[tiab] OR “overweight”[tiab] OR “body mass index”[tiab] OR “quality of life”[tiab] OR “well-being”[tiab] OR “wellbeing”[tiab]) AND (("2000/01/01"[PDat]: "2018/09/30"[PDat]) AND (Japanese[lang] OR English[lang]))
Web of Science	topics: (Japan*) AND title: (“agricultural experience” OR “farming experience” OR “farming workshop” OR “forestry experience” OR “fishery experience” OR “rural experience” OR “food production practice” OR “farm stay” OR agritourism OR “community supported agriculture” OR garden* OR horticulture* OR harvest*) AND (diet* OR food* OR nutri* OR vegetable* OR fruit* OR health* OR exercise OR “physical activity” OR “body weight” OR obesity OR overweight OR “body mass index” OR “quality of life” OR well-being OR wellbeing) AND language: (English OR Japanese) *published from 2000 to 2018
CiNii	(農業体験 OR 農作業体験 OR 農の活動 OR 林業体験 OR 漁業体験 OR 農村体験 OR 生産体験 OR ファームステイ OR アグリツーリズム OR 地域支援型農業 OR 菜園 OR 農園 OR 食農教育 OR 栽培 OR 園芸 OR 収穫) AND (((食 OR 栄養 OR 野菜 OR 果物) AND (摂取 OR 生活 OR 行動 OR 知識 OR 意識 OR 態度 OR 習慣 OR 嗜好)) OR 健康 OR 運動 OR 身体活動 OR 体重 OR 肥満 OR BMI OR “body mass index” OR “quality of life” OR QOL OR ウェルビーイング OR wellbeing OR well-being) *published from 2000 to 2018
ICHUSHI	(農業体験/AL or 農作業体験/AL or 農の活動/AL or 林業体験/AL or 漁業体験/AL or 農村体験/AL or 生産体験/AL or ファームステイ/AL or アグリツーリズム/AL or 地域支援型農業/AL or 菜園/AL or 農園/AL or 食農教育/AL or 栽培/AL or (園芸/TH or 園芸/AL) or 収穫/AL) and ((食生活/TH or 食生活/AL) or (食行動/TH or 食行動/AL) or 食知識/AL or 食意識/AL or 食態度/AL or 食習慣/AL or (食物の嗜好/TH or 食嗜好/AL) or 栄養摂取/AL or 野菜摂取/AL or 果物摂取/AL or (健康/TH or 健康/AL) or 身体運動/TH or 身体活動/AL or (体重/TH or 体重/AL) or (肥満/TH or 肥満/AL) or (BMI/TH or BMI/AL) or “body mass index”/AL or “quality of life”/AL or QOL/AL or ウェルビーイング/AL or wellbeing/AL or well-being/AL) and (DT=2000:2018 and PT=会議録除く)

Table S2. Results of bias risk assessment.

Quality Assessment Tool for Observational Cohort and Cross-Sectional Studies	Kida H. et al. (2018) [21]	Ying G. et al. (2014) [24]	Taniguchi T. et al. (2010) [25]	Taniguchi T. et al. (2010) [26]	Akamatsu R. et al. (2009) [27]	Oura Y. et al. (2009) [28]	Sato K. (2015) [33]	Soga M. et al. (2017) [36]	Machida D. et al. (2017) [37]	Amemiya M. (2012) [38]	Noda T. (2007) [39]
1. Was the research question or objective in this paper clearly stated?	Yes	Yes	Yes	Yes	Yes	Yes	Yes	Yes	Yes	Yes	Yes
2. Was the study population clearly specified and defined?	Yes		Yes	Yes	Yes	Yes	Yes	Yes	Yes	Yes	Yes
3. Was the participation rate of eligible persons at least 50%?	Yes			Yes	Yes	Yes	Yes			Yes	
4. Were all the subjects selected or recruited from the same or similar populations (including the same time period)? Were inclusion and exclusion criteria for being in the study prespecified and applied uniformly to all participants?	Yes	Yes	Yes	Yes	Yes	Yes	Yes	Yes	Yes	Yes	Yes
5. Was a sample size justification, power description, or variance and effect estimates provided?											
6. For the analyses in this paper, were the exposure(s) of interest measured prior to the outcome(s) being measured?											
7. Was the timeframe sufficient so that one could reasonably expect to see an association between exposure and outcome if it existed?							Yes				
8. For exposures that can vary in amount or level, did the study examine different levels of the exposure as related to the outcome (e.g., categories of exposure, or exposure measured as continuous variable)?			Yes	Yes	Yes	Yes		Yes	Yes	Yes	Yes
9. Were the exposure measures (independent variables) clearly defined, valid, reliable, and implemented consistently across all study participants?	Yes	Yes	Yes	Yes	Yes	Yes		Yes	Yes	Yes	
10. Was the exposure(s) assessed more than once over time?	NA	NA	NA	NA	NA	NA		NA	NA	NA	NA
11. Were the outcome measures (dependent variables) clearly defined, valid, reliable, and implemented consistently across all study participants?			Yes		Yes			Yes	Yes	Yes	
12. Were the outcome assessors blinded to the exposure status of participants?											
13. Was loss to follow-up after baseline 20% or less?	NA	NA	NA	NA	NA	NA	NA	NA	NA	NA	NA
14. Were key potential confounding variables measured and adjusted statistically for their impact on the relationship between exposure(s) and outcome(s)?				Yes				Yes	Yes		
Proportion of "Yes" (NA excluded from the denominator)	41.7	25.0	50.0	58.3	58.3	50.0	38.5	58.3	58.3	58.3	33.3

The Study Quality Assessment Tools from the National Institute of Health National Heart, Lung, and Blood Institute

Table S2. *Cont.*

Quality Assessment of Controlled Intervention Studies	Kida H. et al. (2016) [22]	Yoshida T. et al. (2007) [30]	Shimamura M. et al. (2013) [32]
1. Was the study described as randomized, a randomized trial, a randomized clinical trial, or an RCT?			
2. Was the method of randomization adequate (i.e., use of randomly generated assignment)?			
3. Was the treatment allocation concealed (so that assignments could not be predicted)?			
4. Were study participants and providers blinded to treatment group assignment?			
5. Were the people assessing the outcomes blinded to the participants' group assignments?			
6. Were the groups similar at baseline on important characteristics that could affect outcomes (e.g., demographics, risk factors, co-morbid conditions)?	Yes	Yes	
7. Was the overall drop-out rate from the study at endpoint 20% or lower of the number allocated to treatment?			Yes
8. Was the differential drop-out rate (between treatment groups) at endpoint 15 percentage points or lower?	Yes		Yes
9. Was there high adherence to the intervention protocols for each treatment group?	Yes		
10. Were other interventions avoided or similar in the groups (e.g., similar background treatments)?			
11. Were outcomes assessed using valid and reliable measures, implemented consistently across all study participants?		Yes	
12. Did the authors report that the sample size was sufficiently large to be able to detect a difference in the main outcome between groups with at least 80% power?			
13. Were outcomes reported or subgroups analyzed prespecified (i.e., identified before analyses were conducted)?	Yes	Yes	Yes
14. Were all randomized participants analyzed in the group to which they were originally assigned, i.e., did they use an intention-to-treat analysis?	Yes	Yes	Yes
Proportion of "Yes" (NA excluded from the denominator)	35.7	28.6	28.6

The Study Quality Assessment Tools from the National Institute of Health National Heart, Lung, and Blood Institute

Table S2. *Cont.*

Quality Assessment Tool for Before-After (Pre–Post) Studies with no Control Group	Kida H. et al. (2016) [22]	Kanno Y. et al. (2011) [23]	Ying G. et al. (2014) [24]	Yamada I. (2008) [29]	Yamamoto T. (2008) [31]	Tsuchihashi Y. (2010) [34]	Otake M. et al. (2010) [35]
1. Was the study question or objective clearly stated?	Yes	Yes	Yes	Yes	Yes	Yes	Yes
2. Were eligibility/selection criteria for the study population prespecified and clearly described?	Yes	Yes		Yes	Yes	Yes	Yes
3. Were the participants in the study representative of those who would be eligible for the test/service/intervention in the general or clinical population of interest?	Yes	Yes	Yes	Yes	Yes	Yes	
4. Were all eligible participants that met the prespecified entry criteria enrolled?	Yes	Yes		Yes	Yes		
5. Was the sample size sufficiently large to provide confidence in the findings?							
6. Was the test/service/intervention clearly described and delivered consistently across the study population?	Yes	Yes	Yes	Yes		Yes	Yes
7. Were the outcome measures prespecified, clearly defined, valid, reliable, and assessed consistently across all study participants?				Yes	Yes	Yes	Yes
8. Were the people assessing the outcomes blinded to the participants' exposures/interventions?							
9. Was the loss to follow-up after baseline 20% or less? Were those lost to follow-up accounted for in the analysis?							Yes
10. Did the statistical methods examine changes in outcome measures from before to after the intervention? Were statistical tests done that provided p values for the pre-to-post changes?	Yes	Yes	Yes	Yes	Yes	Yes	Yes
11. Were outcome measures of interest taken multiple times before the intervention and multiple times after the intervention (i.e., did they use an interrupted time-series design)?	Yes	Yes	Yes	Yes	Yes	Yes	Yes
12. If the intervention was conducted at a group level (e.g., a whole hospital, a community, etc.) did the statistical analysis take into account the use of individual-level data to determine effects at the group level?	NA	NA	NA	NA	NA	NA	NA
Proportion of "Yes" (NA excluded from the denominator)	63.6	63.6	45.5	72.7	63.6	63.6	63.6

The Study Quality Assessment Tools from the National Institute of Health National Heart, Lung, and Blood Institute

PRISMA 2009 Checklist

Section/topic	#	Checklist item	Reported on page #
**TITLE**			
Title	1	Identify the report as a systematic review, meta-analysis, or both.	1
**ABSTRACT**			
Structured summary	2	Provide a structured summary including, as applicable: background; objectives; data sources; study eligibility criteria, participants, and interventions; study appraisal and synthesis methods; results; limitations; conclusions and implications of key findings; systematic review registration number.	1
**INTRODUCTION**			
Rationale	3	Describe the rationale for the review in the context of what is already known.	1-2
Objectives	4	Provide an explicit statement of questions being addressed with reference to participants, interventions, comparisons, outcomes, and study design (PICOS).	2
**METHODS**			
Protocol and registration	5	Indicate if a review protocol exists, if and where it can be accessed (e.g., Web address), and, if available, provide registration information including registration number.	2
Eligibility criteria	6	Specify study characteristics (e.g., PICOS, length of follow-up) and report characteristics (e.g., years considered, language, publication status) used as criteria for eligibility, giving rationale.	2-3
Information sources	7	Describe all information sources (e.g., databases with dates of coverage, contact with study authors to identify additional studies) in the search and date last searched.	2-3
Search	8	Present full electronic search strategy for at least one database, including any limits used, such that it could be repeated.	2-3, Suppl.
Study selection	9	State the process for selecting studies (i.e., screening, eligibility, included in systematic review, and, if applicable, included in the meta-analysis).	2-3
Data collection process	10	Describe method of data extraction from reports (e.g., piloted forms, independently, in duplicate) and any processes for obtaining and confirming data from investigators.	2-3
Data items	11	List and define all variables for which data were sought (e.g., PICOS, funding sources) and any assumptions and simplifications made.	2-3
Risk of bias in individual studies	12	Describe methods used for assessing risk of bias of individual studies (including specification of whether this was done at the study or outcome level), and how this information is to be used in any data synthesis.	3
Summary measures	13	State the principal summary measures (e.g., risk ratio, difference in means).	3
Synthesis of results	14	Describe the methods of handling data and combining results of studies, if done, including measures of consistency (e.g., I^2) for each meta-analysis.	3

Section/topic	#	Checklist item	Reported on page #
Risk of bias across studies	15	Specify any assessment of risk of bias that may affect the cumulative evidence (e.g., publication bias, selective reporting within studies).	none
Additional analyses	16	Describe methods of additional analyses (e.g., sensitivity or subgroup analyses, meta-regression), if done, indicating which were pre-specified.	none
**RESULTS**			
Study selection	17	Give numbers of studies screened, assessed for eligibility, and included in the review, with reasons for exclusions at each stage, ideally with a flow diagram.	3 - 4
Study characteristics	18	For each study, present characteristics for which data were extracted (e.g., study size, PICOS, follow-up period) and provide the citations.	4 - 11
Risk of bias within studies	19	Present data on risk of bias of each study and, if available, any outcome level assessment (see item 12).	6 – 12, Suppl.
Results of individual studies	20	For all outcomes considered (benefits or harms), present, for each study: (a) simple summary data for each intervention group (b) effect estimates and confidence intervals, ideally with a forest plot.	4 - 11
Synthesis of results	21	Present results of each meta-analysis done, including confidence intervals and measures of consistency.	none
Risk of bias across studies	22	Present results of any assessment of risk of bias across studies (see Item 15).	none
Additional analysis	23	Give results of additional analyses, if done (e.g., sensitivity or subgroup analyses, meta-regression [see Item 16]).	none
**DISCUSSION**			
Summary of evidence	24	Summarize the main findings including the strength of evidence for each main outcome; consider their relevance to key groups (e.g., healthcare providers, users, and policy makers).	12 - 15
Limitations	25	Discuss limitations at study and outcome level (e.g., risk of bias), and at review-level (e.g., incomplete retrieval of identified research, reporting bias).	15
Conclusions	26	Provide a general interpretation of the results in the context of other evidence, and implications for future research.	15
**FUNDING**			
Funding	27	Describe sources of funding for the systematic review and other support (e.g., supply of data); role of funders for the systematic review.	15
